# Navigating through recent evidence on locoregional breast cancer radiotherapy: an initiative by the scientific association of Swiss radiation oncology

**DOI:** 10.1007/s00066-024-02332-5

**Published:** 2024-12-06

**Authors:** Pelagia G. Tsoutsou, Anna-Lena Eberhardt, Günther Gruber, Guido Henke, Wendy Jeannerret-Sozzi, Claudia Linsenmeier, Kristina Lössl, Maria-Carla Valli, Walter P. Weber, Kathrin Zaugg, Khalil Zaman, Daniel Zwahlen

**Affiliations:** 1 Radiation Oncology Department, Geneva University Hospitals (HUG) and Faculty of Medicine, University of Geneva (UNIGE), Avenue de la Roseraie 53, 1205 Geneva, Switzerland; 2 Radiation Oncology Department, University Hospital Basel (USB), Basel, Switzerland; 3 Radiation Oncology Department, Hirslanden Zurich, Zurich, Switzerland; 4 Radiation Oncology Department, Team Radiology Plus, Münsterlingen, Switzerland; 5 Radiation Oncology Department, Lausanne University Hospital (CHUV), Lausanne, Switzerland; 6 Radiation Oncology Department, Zurich University Hospital (USZ), Zurich, Switzerland; 7 Radiation Oncology Department, Inselsital, Bern University Hospital, University of Bern, Bern, Switzerland; 8 Radiation Oncology Department, Bellinzona Hospital (IOSI), Bellinzona, Switzerland; 9 Breast Cancer Surgery Department, Basel University Hospital (USB), Basel, Switzerland; 10 Radiation Oncology Department, Stadtspital Zürich, Zürich, Switzerland; 11 Medical Oncology Service, Lausanne University Hospital CHUV, Lausanne, Switzerland; 12 Radiation Oncology Department, Winterthur Cantonal Hospital, Winterthur, Switzerland; 13 SASRO, https://www.sasro.ch/

**Keywords:** Education, Evidence-based practice, Guidelines, Evidence, Tool

## Abstract

**Purpose:**

Breast cancer (BC) is the most prevalent cancer in women and radiotherapy (RT) is an integral part of its treatment. High-level evidence guides clinical decisions, but given the abundance of guidelines, a need to navigate within the evidence has been identified by the board of the Scientific Association of Swiss Radiation Oncology (SASRO). A pilot project was initiated aiming to create an overview of recent clinically relevant evidence for BC RT, to make it easily available to (radiation) oncologists and radiation oncologists in training.

**Methods:**

A panel of 10 radiation oncology experts for BC RT, one expert in BC surgery, and one expert in BC medical oncology critically reviewed the relevant literature. The panel comprehensively represented different geographical regions of Switzerland as well as university, cantonal, and private institutions. We sought to create a consensual overview of the most relevant questions in BC RT today, accompanied by the most recent and relevant available evidence.

**Results:**

From January 2023 to January 2024, the panel met four times to review and work on an initial draft. The final draft was reviewed and accepted by all panelists. We hereby publish this work to make it available to international audiences. After publication, the work will be made available to all SASRO members on the SASRO website. This work is to be updated every 2 years.

**Conclusion:**

The identified need was addressed with a successful pilot project and will be further expanded upon in other tumor pathologies.

## Introduction

Breast cancer (BC) is the most common cancer in women [1]. Tremendous progress has been achieved in its treatment through recent decades, improving disease-free (DFS) and overall survival (OS) [2]. Treatment of early BC is multimodal: it addresses both the local and systemic components of the disease with surgery with or without radiotherapy (RT) for the former and systemic treatments (hormone therapy, chemotherapy, targeted treatments, and immunotherapy) for the latter [3, 4]. RT is an essential modality of locoregional management because it has permitted a de-escalation of surgery in breast and axilla [5] and provides local control and OS benefits after lumpectomy and even mastectomy in higher-risk patients [6, 7].

The vast amount of published evidence on BC RT merits putting it into context, so that it is readily available for evidence-based decisions in the routine treatment of the individual cancer patient. Indeed, in modern BC RT, several evidence-based options exist. Their overview and understanding can promote quality in shared or multidisciplinary decision-making and represents the scope of this work.

The Scientific Association of Swiss Radiation Oncology (SASRO) is the national scientific multidisciplinary society in radiation oncology. Given its mission to promote education in the discipline within Switzerland, a pilot project was initiated by the SASRO board to address this need and is presented in this manuscript.

This educational SASRO initiative aims to provide an updated, clinically relevant and comprehensive background of existing evidence on radiation oncology treatment of BC. It is not a narrative review of the literature, nor does this work aim to give specific recommendations for clinical practice, since this has already been undertaken within the work presented here. The goal of this work is to make available all the relevant information for evidence-based radiation oncology treatment of BC, so that clinicians can decide for themselves which guideline to adopt and which discussion on a grey zone they are going to adhere to in their clinical practice.

## Methods

This work was undertaken through January 2023 to January 2024. The SASRO board assigned the tasks of conceptualization, methodology, and final manuscript drafting to one of its members (PT). A first draft including methodology was presented to the SASRO board at the 2023 SASRO board retreat. Upon discussion and board approval, a panel of 9 additional BC radiation oncology experts for BC treatment, representing academic, public cantonal, and private centers within Switzerland and distributed among Swiss regions (German, French and Italian speaking), was defined. An expert BC surgeon (WW) and an expert BC medical oncologist (KhZ) were included in the panel to provide insights from these relevant disciplines. The panel met four times, approximately every 3 months, and worked on the initial draft. Input from all panelists was critically discussed and integrated in the final draft based on consensus. After review, the final draft of the manuscript was approved by the panel and submitted to the SASRO board for final approval at the 2024 SASRO board retreat. This draft is to be posted on the SASRO website after publication of the present manuscript, submitted to the official journal of SASRO, *Strahlentherapie Onkologie*.

Breast cancer is a disease with much evidence and subtlety, and this work aimed to highlight all the available options and guidance without replacing existing guidelines. The project was defined as such by SASRO and aims to reflect the Swiss mentality of consensus and acknowledgement of possible multiple different views that can be applicable.

The recommendations will be updated by the panel every 2 years. After completion of this pilot project, future projects of the SASRO board include undertaking this work for other tumor pathologies (e.g., head and neck and/or prostate cancer).

## Results

### BC RT indications

#### Standard recommendations for BC RT indications

include the statements that every decision on BC RT must be undertaken within a BC tumor board, preferably within a certified BC center. All patients with BC should have access to comprehensive cancer care [8]. Participating in clinical trials improves patients’ outcomes, and enrolment should be encouraged by BC tumor boards and BC radiation oncologists. Minimum requested work-up for BC diagnosis is described in the NCCN (National Comprehensive Cancer Network) and ESMO (European Society for Medical Oncology) guidelines. The most recent, essential guidelines for BC RT have been identified and are presented in Table 1. The number of radiation oncologists (ROs)/specialists out of all specialists participating in the guidelines/consensus is also illustrated.

**Table 1:** Reference guidelines for early-stage primary breast cancer

Guideline	Description	Reference	ROs/specialists
NCCN v 5.2023	Decision trees for BC [14]	https://www.nccn.org/login?ReturnURL=https://www.nccn.org/professionals/physician_gls/pdf/breast.pdf	4/44
St Gallen	Expert consensus [15]	Curigliano G, Burstein HJ, Gnant M et al. *Understanding breast cancer complexity to improve patient outcomes: The St. Gallen International Consensus Conference for the Primary Therapy of Individuals with Early Breast Cancer*. An Oncol 2023 34;11: 970–986	3/61
ESMO	Guideline [16]	S. Loibl, F. André, T. Bachelot et al, on behalf of the ESMO Guidelines Committee. *Early Breast Cancer: ESMO Clinical Practice Guidelines for diagnosis, treatment and follow-up.* Annals of Oncology (2024), 10.1016/j.annonc.2023.11.016	1/8
ASCO	Specific guideline on axilla management [17]	Brackstone M, Baldassarre FG, Perera FE et al. *Management of the Axilla in Early-Stage Breast Cancer: Ontario Health (Cancer Care Ontario) and ASCO Guideline.* JCO 2021 Sep 20;39(27):3056–3082	3/14
SSO-ASTRO-ASCO	Consensus on margins in DCIS [18]	Morrow M, Van Zee KJ, Solin LJ et al. *Society of Surgical Oncology—American Society for Radiation Oncology—American Society of Clinical Oncology Consensus Guideline on Margins for Breast-Conserving Surgery with Whole-Breast Irradiation in Ductal Carcinoma in Situ.* J Clin Oncol. 2016 34:33, 4040–4046	4/13
ASCO	Guideline on post-mastectomy RT for early-stage BC [19]	Recht A, Comen EA, Fine RE et al. *Postmastectomy Radiotherapy: An American Society of Clinical Oncology, American Society for Radiation Oncology, and Society of Surgical Oncology Focused Guideline Update.* JCO 2016 34:36, 4431–4442	6/17
ESTRO	Recommendations on IORT [20]	Fastner G, Gaisberger C, Kaiser J et al. *ESTRO IORT Task Force/ACROP recommendations for intraoperative radiation therapy with electrons (IOERT) in breast cancer.* Radiother Oncol. 2020;149:150–157	NA
ESTRO	Guideline on brachytherapy [21]	Strnad V, Major T, Polgar C et al. *ESTRO-ACROP guideline: Interstitial multi-catheter breast brachytherapy as Accelerated Partial Breast Irradiation alone or as boost—GEC-ESTRO Breast Cancer Working Group practical recommendations.* Radiother Oncol. 2018;128(3):411–420	NA
ESTRO	Consensus on fractionation [22]	Meattini I, Becherini C, Boersma L, et al. *European Society for Radiotherapy and Oncology Advisory Committee in Radiation Oncology Practice consensus recommendations on patient selection and dose and fractionation for external beam radiotherapy in early breast cancer.* Lancet Oncol. 2022 Jan;23(1):e21–e31. 10.1016/S1470-2045(21)00539-8. PMID: 34973228	14/22
ESTRO-endorsed	Consensus on association of BC RT and drugs [23]	Meattini I, Becherini C, Caini S et al.; Consensus Panellist Group. *International multidisciplinary consensus on the integration of radiotherapy with new systemic treatments for breast cancer: European Society for Radiotherapy and Oncology (ESTRO)-endorsed recommendations.* Lancet Oncol. 2024 Feb;25(2):e73–e83. 10.1016/S1470-2045(23)00534-X. PMID: 38301705	8/19
ASTRO	Guideline on PBI [12]	Shaitelman SF, Anderson BM, Arthur DW, et al. *Partial Breast Irradiation for Patients With Early-Stage Invasive Breast Cancer or Ductal Carcinoma In Situ: An ASTRO Clinical Practice Guideline.* Pract Radiat Oncol. 2024 Mar-Apr;14(2):112–132	All

*DCIS* ductal carcinoma in situ, *IORT* intraoperative radiotherapy, *PBI* partial breast irradiation

Emerging disease settings include oligometastatic disease, for which appropriate imaging and treatments are clinical research priorities [9]—a setting where the optimal treatment approach remains to be defined and should be discussed in a multidisciplinary setting. Emerging indications include partial breast irradiation (PBI), representing a standard option for early, low-risk breast cancer [10], with preferred modalities being external-beam RT with CT planning or brachytherapy [11]. Intraoperative radiotherapy (IORT) remains a possible option in carefully selected patients with very-low-risk BC, such as luminal A T1a‑b N0 tumors and elderly patients with comorbidities, although most recent guidelines advise against more generalized use for low-risk patients [12]. Low-risk BC or ductal carcinoma in situ (DCIS) represents a personalized setting where adjuvant treatment trade-offs should be discussed within a shared decision-making context [13].

#### Grey zones in BC RT indications

have been identified and discussed. We recognized *regional BC RT*, which has long been a matter of debate, as a pertaining grey zone, despite the recent Oxford meta-analysis confirming small benefits of regional BC RT for distant control and BC-related mortality, with no negative impact on non-BC mortality within modern trials. The magnitude of the benefit is larger in higher-risk patients [24]. Treatment of the axilla and the associations of surgery and RT in the era of de-escalation of the latter remain challenging and are regularly addressed in multidisciplinary panels of BC specialists [25]. As this field is evolving and the object of research underway, results of ongoing trials, such as MA.39 (NCT03488693), will permit better identification of risk factors for addition or omission of regional RT by integrating biology. Of note, the indicated irradiation volumes when regional RT is performed also remain a matter of debate, as the pivotal studies, such as ACOSOG Z0011 [26], AMAROS [27], MA.20 [28], and EORTC-IMC [29], all used slightly different irradiation volumes.

#### Post-M RT (PMRT)

another possible grey zone, especially when intermediate-risk stages, such as pT1‑2 pN1, are concerned. For these stages, especially after axillary lymph node dissection (ALND), the benefit of PMRT with modern systemic treatments can be challenged, as discussed in the ASCO guidelines on PMRT [19]—and this despite the Oxford meta-analysis that advises PMRT after ALND for any node-positive patient [7]. As surgery is de-escalated and systemic treatments evolve and improve outcomes, these indications can be debated in more modern contexts. Despite this, it must be kept in mind that improved systemic treatments will probably make BC RT even more efficient and relevant [30].

#### Irradiation after neoadjuvant chemotherapy (NACT)

remains another grey zone of possible de-escalation. Most recent guidelines advise the consideration of initial nodal involvement for decision-making regarding regional RT. For selected cases with cT1‑2 cN1 disease transforming into ypN0 after NACT and ALND, de-escalation of RT emerged as a potentially safe consideration after the publication of the prospective registry study RAPCHEM [31]. However, the panel noted that this is a prospective registry study, where around 80% of patients received ALND after NACT, a practice that is now being abandoned in cases of ycN0. The study is highly relevant as proof of principle but not sufficient for practice change. The recent presentation of NSAPB-51 at the San Antonio Breast Cancer Symposium (SABCS) 2023 is a practice changing phase III study showing no benefit of regional RT in patients with cT1‑3 cN1 BC converting into ypN0 [32].

Another grey zone in the field of axillary management and work-up is described in ASCO guidelines [19]. Omission of sentinel lymph node dissection (SLND) in low-risk cN0 BC becomes an evidence-based option, given the recent publication of the SOUND trial [33]. The panel recognized that this is a fast-moving field, with more data expected in the coming years.

Finally, for a detailed discussion on modern BC RT controversies, the *JCO special issue on locoregional management of BC *that appeared in May 2020 remains a precious resource [34]. BC RT indications as standard options recognized by the panel are presented in Table 2.

**Table 2:** Volumes, doses, and fractionation for breast cancer radiotherapy: indicative options

Indication	Irradiation	Irradiation doses (Gy)	Fractionation (Gy/fr)	Number of fractions
	**BCS**			
*DCIS*	Low-risk DCIS: RT indication as shared decision with patient Breast +/− boost tumor bed Boost: shared decision with patient	Breast 40.5 + SIB 48	2.7/3.2	15
*Reading*	Boost for DCIS [35]—SIB [36]			
*pT1pN0* *Preferably: luminal A* *>* *60y*	Partial breast irradiation tumor bed	PTV 30*	6	5; every other day
*pT1pN0, luminal, >* *40y*	HDR brachytherapy	Tumor bed + 20 mm 30.1	7	In 5 days; twice daily
*Margin* *>* *2* *mm*		Tumor bed + 20 mm 32	8	
	PDR brachytherapy	Tumor bed + 20 mm 50 , pulses 0.6–0.8 Gy/h	–	
*Reading*	External beam RT PBI [37]			
*pT1‑2 pN0*	Breast +/− boost tumor bed	Breast 40.5/48 SIB	2.7‑3.2	15
	Boost: shared decision with patient after 50y	Breast 40.5 +/− boost 10.8–13.5	2.7	15 + 5
	Moderate hypofractionation preferable	Breast 36/partial breast 40/48 tumor bed	2.4/2.67/3.2	15
	Elderly: extreme hypofractionation an option	Breast 42.56 + boost 10.64	2.66	16 + 4
		Breast 50/SIB 60	2	25
		Breast 28.5	5.7	5; once weekly
		Breast 26	5.2	5 within a week
*Reading*	FAST [38] and FAST FORWARD [39]			
*pN1 and SLND*	Breast +/− boost tumor bed	Breast 50/SIB 60	2–2.4	25
	Boost: shared decision with patient after 50y	Regional nodes: 45–50	1.8–2	25
	+ axilla I–III, consider IV, consider IMC	–	–	15
	Consider regional RT if 1–2 nodes positive, no ECE	–	–	15
	Consider IMC if QE	–	–	–
	Local RT according to ACOSOG Z0011 is an option for low-risk patients	–	–	–
	Hypofractionation an option	Breast 36/partial breast 40/48 tumor bed	2.4/2.67/3.2	15
		Lymph nodes 40	2.67	15
		Breast and regional nodes 40.5	2.7	15
		Breast and regional nodes 42.56	2.66	16
*pN2/pN3*	Breast +/− boost tumor bed	Breast 50+ boost 10–16 or	2	25–33
	Boost: shared decision with patient after 50y	Breast 50+ SIB 60	2–2.4	25
	+ axilla III, IV, IMC	Regional nodes 50	2	25
	Consider boost in unresected regional disease	55 Gy	2.2	25
	Hypofractionation an option	Breast and regional nodes 40.5	2.7	15
		Breast and regional nodes 42.56	2.66	16
	**PMRT**			
*pT3pN0*	Chest wall	Chest wall 50	2	25
	Hypofractionation an option if no immediate reconstruction	Chest wall 40.5	2.7	15
		Chest wall 42.56	2.66	16
*pT4*	Chest wall +/− boost scar	Chest wall 50 + boost 10–16	2	25–33
	Bolus if skin invasion	–	–	–
	Consider regional RT	–	–	–
	Hypofractionation an option if no immediate reconstruction	Chest wall 40.5 +/− boost 10.8–13.5	2.7	15–20
		Chest wall 42.56 +/− boost 10.64	2.66	16–20
*pN1 and SLND*	Chest wall + axilla I–III, consider IV, consider IMC	Chest wall 50	2	25
		Regional nodes 45–50	1.8–2	25
	Consider IMC if QE	–	–	–
	Hypofractionation in elderly patient if no immediate reconstruction	Chest wall and regional nodes 40.5	2.7	15
		Chest wall and regional nodes 42.56	2.66	16
*pN2/pN3*	Chest wall + axilla III, IV, IMC	Chest wall + regional nodes 50	2	25
	Consider boost in unresected regional disease	55	2.2	25
	Hypofractionation in elderly patients	Chest wall and regional nodes 40.5	2.7	15
		Chest wall and regional nodes 42.56	2.66	16

## BC RT technique, irradiation volumes, dosimetry, and fractionation

In terms of the *BC RT technique*, the panel recognized that the optimum available technique, providing coverage of targets and protection of organs at risk (OARs), including consideration of the risk of secondary cancer, should be used; 3D and/or intensity-modulated techniques are valid options in this setting. The use of deep-inspiration breath hold (DIBH) to protect the heart/lung/liver, when feasible and advantageous from a dosimetric point of view, especially for left-sided tumors, was strongly supported [40, 41]. Treatment in the prone position is an option to decrease acute toxicity for selected cases, especially in patients with voluminous breasts and the indication of whole-breast irradiation (WBI) only [42]. The use of bolus should be avoided in PMRT, unless in case of skin involvement of the disease [43].

In terms of *target volume delineation*, the major ESTRO-endorsed guideline is commonly used [44], with a note of the panelists to remind that these volumes are appropriate for early BC. Enlarged target volumes can be considered for locally advanced BC (LABC) [45]. An alternative atlas with enlarged target volumes was used for the RADCOMP study and can be found at the RTOG site [46]. An ESTRO-endorsed atlas for clinical volume delineation after PMRT and immediate reconstruction with implants can be considered, but clinical experience and adoption by the panelists remains limited [47]. Finally, artificial intelligence (AI) solutions for contouring, preferably not atlas based, might be useful and are commonly used by some members of the panel.

In terms of tumor bed delineation, surgical clips are strongly advised for boost delineation and PBI, especially in the context of oncoplasty. PBI target volume delineation strongly depends on the technique used. For both WBI and PBI, peer review of volumes and/or dosimetry is strongly advised.

In terms of *BC RT fractionation*, multiple fractionation regimens are supported by high-level evidence. A recent ESTRO-endorsed consensus was published on appropriate fractionation options [22]. Given the heterogeneity of fractionation regimens adopted in Switzerland and the specific availability of resources in the country, the panel decided to provide a comprehensive table of all available studies on fractionation regimens tested (Table 3).

**Table 3:** Overview of major phase III fractionation studies

Study Publication year	Age	Disease stage	No. patients	Surgery type (BCS/M)	RT regimen (no. factions × dose/fraction [Gy/fr])	FU (years)	Endpoint	Conclusion
Royal Marsden [48] 2006	< 75 years	pT1-3a pN0‑1 M0	1410	BCS	25 × 2 vs. 13 × 3.3 vs. 13 × 3	9.7	Late change in breast appearance	α/β late change in breast = 3.6 α/β breast induration = 3.1 α/β breast cancer = 4
START A [49] 2008	Any 5% < 40 years	pT1-3a pN0‑1 M0	2236	BCS: 84% M: 16%	25 × 2 vs. 13 × 3.2 in 5 weeks vs. 13 × 3 in 5 weeks	5.1	LR Late normal tissue effects QoL	Winner: 13 × 3.2 in 5 weeks
START B [50] 2008	Any 5% < 40 years	pT1-3a pN0‑1 M0	2215	BCS: 92% M: 8%	25 × 2 vs. 15 × 2.67	6	LR Late normal tissue effects QoL	Winner: 15 × 2.67
Canadian [51] 2010	Any 25%< 50 years	pT1‑2 pN0 M0	1234	BCS	25 × 2 vs. 16 × 2.66	10	LR	Non-inferiority 16 × 2.66
IMPORT LOW [52] 2017	> 50 years	pT1‑2 pN0‑1 M0 T < 3 cm	2018	BCS	15 × 2.67 WBI vs. 15 × 2.4 WBI + 15 × 2.67 PBI vs. 15 × 2.67 PBI	6	LR	Non-inferiority 15 × 2.4 WBI + 15 × 2.67 PBI vs. 15 × 2.67 PBI
Chinese [53] 2019	18–75 years 51%< 50 years	pT3-4pN2‑3 M0	820	M	25 × 2 vs. 15 × 2.9	4.9	LRR	Non-inferiority 15 × 2.9
Chinese [54] 2020	18–70 years 40%< 45 years	pT1-2pN0‑3 M0	734	BCS	25 × 2 + 5 × 2 boost vs. 15 × 2.9 + 3 × 2.9 boost	6.1	5‑year LRR	Non-inferiority 15 × 2.9 + 3 × 2.9 boost
FAST [38] 2020	≥ 50 years	pT1‑2 pN0 M0	915	BCS	25 × 2 vs. 5 × 6 once weekly vs. 5 × 5.7 once weekly	10	Change in photographic appearance at 2 and 5 years	Winner: 5 × 5.7 once weekly
DBCG Hypo [55] 2020	> 40 years	pT1‑2 pN0 pM0 and DCIS (13%)	1854	BCS	25 × 2 vs. 15 × 2.67	7.26	3‑year Gr 2–3 breast induration with non-inferiority LR	Non-inferiority 15 × 2.67
FAST FORWARD [56] 2020	> 18 years 1% < 40 years	pT1-3a pN0‑1 M0	4096	BCS 93% M 7% Regional substudy:11% patients/no IMC	15 × 2.67 vs. 5 × 5.4 vs. 5 × 5.2	5.95	LR	Non-inferiority 5 × 5.2
BIG 3‑07/TROG 07.01 [35] 2022	> 18 years	Non-low-risk DCIS	1608	BCS	25 × 2 vs. 16 × 2.66 R boost vs no boost (8 × 2)	6.6	Time to LR	Winner: 16 × 2.66 Boost: reduced LR but increased Gr 2 toxicity
SKAGEN Unpublished Offerssen VB, ESTRO 2022	> 18 years	pT1‑3 pN0‑3 M0 Regional nodes RT	2879	BCS: 52% M: 48%	25 × 2 WBI +/− SIB 25 × 2.28/25 × 2.52 15 × 2.67 WBI +/−SIB 15 × 3.05/15 × 3.48 SIB: 16%	2	3‑year lymphedema	Non-inferiority 15 × 2.67 WBI +/− SIB 15 × 3.05/15 × 3.48
HypoG01: UNICANCER Unpublished Rivera S, ESTRO 2023	> 18 years	pT1‑3 pN0‑3 M0 Regional nodes RT	1265	BCS: 55% M: 45% ALND: 82.8%	25 × 2 15 × 2.67 Boost or SIB: 48.8%	3.1	3‑year lymphedema	Non-inferiority 15 × 2.67
IMPORT HIGH [57] 2023	Min 45 years	pT1-3pN0-3a M0	2617	BCS	15 × 2.67 WBI + 8 × 2 boost TB 15 × 2.4 WBI +15 × 2.67 PBI + 15 × 3.2 TB vs. 15 × 2.4 WBI + 15 × 2.67 PBI + 15 × 3.53 TB	6.2	LR	Dose escalation not advantageous
GEC-ESTRO interstitial brachytherapy [11]	> 40 years	pT1 (89%), pT2 (11%) DCIS 4%	1328	BCS	25 × 2 WBI + 5 × 2 boost vs. Tumor bed + 20 mm 30.1 Gy HDR Tumor bed + 20 mm 32 Gy HDR Tumor bed + 20 mm 50 Gy, pulses 0.6–0.8 Gy/h, PDR	10.36	LR	Non-inferiority APBI

*APBI* accelerated partial breast irradiation, *BCS* breast-conserving surgery, *CW* chest wall, *ECE* extracapsular extension, *EQ* external quadrant, *IMC* internal mammary chain, *LR* local recurrence, *LRR* locoregional recurrence, *M* mastectomy, *PBI* partial breast irradiation, *PMRT* postmastectomy radiotherapy, *SIB* simultaneously integrated boost, *T* tumor bed, *y* year, *w* weeks, *WBI* whole-breast irradiation

## Specific topics

The panel wished to address specific topics that are often debated in multidisciplinary tumor boards.

One such topic is the *omission of RT in low-risk BC in elderly patients, *which was recognized as an option in patients receiving adjuvant endocrine therapy, with recent relevant literature [58–60] discussed in the *New England Journal of Medicine* [61, 62]. However, when endocrine therapy is not considered, BC RT can be a valuable option, as discussed by Naoum [97] or Ward and colleagues [63]. An ongoing study, EUROPA (NCT04134598) [64], will ultimately address the trade-off in quality of life between a few fractions of PBI versus 5 years of endocrine therapy, as discussed in the *New England Journal of Medicine* [61, 62]. However, when endocrine therapy is not considered or feasible, BC RT can be a valuable option, as discussed by Naoum [97] or Ward and colleagues [63].

*BC RT in the context of autoimmune disease *is another specific topic for which evidence is very scarce. Modern literature suggests that BC RT toxicity is acceptable and might be slightly increased in patients with autoimmune disease as compared to those without, especially when their autoimmune disease is active, with some recent evidence emerging on the use of hypofractionation [65] or [66] hypofractionation/PBI in this context.

*BC RT in the setting of patients with a genetic predisposition to cancer *has been identified as another specific topic, meriting review of the evidence. The only absolute contraindication for BC RT remains the Li-Fraumeni syndrome, which is associated with a high risk of secondary malignancies after RT [67–69]. For patients with BC who are *BRCA mutation carriers, *BC RT is as safe as in the general population [70–72].

The *combination of BC RT with new drugs *established in the adjuvant disease setting is an emerging specific topic of major importance, treated in several recent comprehensive reviews [73], the most recent being ESTRO endorsed [23]. As far as BC RT and the current adjuvant *anti-HER‑2 targeted therapies *are concerned, their combination concurrently with BC RT seems to be safe, especially with new irradiation techniques sparing the heart [74, 75]. Regarding the concomitant use of BC RT and *T‑DM1, *a slight increase in pneumonitis has been noted in the adjuvant setting, with overall safety being considered acceptable, but with caution needed when irradiating intracranial sites in the metastatic setting [76]. Regarding the concurrent use of BC RT and *trastuzumab deruxtecan, *it is advised not to combine them concomitantly in the adjuvant setting [75]. *Immunotherapy* with pembrolizumab and atezolizumab can be given concomitantly with adjuvant BC RT, although few studies are available on this and mainly concern the metastatic setting [77].

Regarding combinations of BC RT and *CDK4/6 inhibitors, *in the absence of sufficient data, caution is needed in the adjuvant setting. Concomitant administration seems to be safe in the metastatic setting [78]. The combination with *olaparib *in high-risk TNBC is based on preliminary evidence that is reassuring [79]. In the pivotal phase III OlympiA trial, BC RT and olaparib were administered sequentially [80] and it should be stated that the recent ESTRO consensus recommends against concurrent use [23]. The combination of BC RT and *capecitabine *in the setting of advanced BC has been tested in a phase II study [81], while in the adjuvant setting, capecitabine is provided after the completion of adjuvant BC RT, as per the CREATE.X study [82]. The combination of stereotactic body RT (SBRT) and drugs in the oligometastatic setting was the object of a literature review and an expert consensus within the OligoCare consortium [83].

*Re-irradiation for local recurrence *has been discussed as another specific topic [84–86]. Indeed, breast preservation with partial breast re-irradiation is emerging as an option for selected patients with late low-risk local recurrence after initial breast-conserving surgery and adjuvant WBI [87, 88]. Brachytherapy has been the most commonly used technique for partial breast re-irradiation, with good oncological outcomes and tolerance [89, 90]. For chest wall recurrences and the need to re-irradiate in the adjuvant or definitive setting, combination with hyperthermia is an interesting option, available in a number of centers in Switzerland today, which should be considered [91, 92]. A recent resource on re-irradiation globally was published as an ESTRO-EORTC expert consensus [93].

Finally, *integrative medicine tools *are being incorporated in international guidelines, such as the ASCO, and these resources can be of use to the clinician [94–96].

## Access to clinical studies in Switzerland

The panel also advised to include a list of all resources for open studies in Switzerland. These can be found on the websites of the Swiss Group for Clinical Cancer Research (SAKK; https://www.sakk.ch/en/trials); the European Organization for Research and Treatment of Cancer (EORTC; https://www.eortc.org/clinical-trials-database/); the International Breast Cancer Study Group (IBCSG; https://www.ibcsg.org/en/patients-professionals/clinical-trials/open-trials), which is specific for clinical and translational research in BC; and the European Thoracic Oncology Platform (ETOP; https://www.etop-eu.org/index.php?option=com_content&view=category&id=408&Itemid=195), which is specific for clinical and translational research in lung cancer.

## Conclusion

As part of a pilot project initiated by the SASRO board, we completed a comprehensive overview with a brief discussion of the most recent and clinically relevant evidence for BC RT clinical care. This tool could contribute to quality improvement of radiation oncology decision-making within a multidisciplinary setting and could additionally be used for teaching purposes within the radiation oncology community. The present work is evidence based but has permitted integration of BC experts’ views, mainly radiation oncologists but also a BC surgeon and a medical oncologist, all around Switzerland, reflecting modern clinical care in radiation oncology in the BC setting in the country.

## References

[1] Siegel RL, Giaquinto AN, Jemal A (2024) Cancer statistics, 2024. CA A Cancer J Clinicians 74(1):12–49 10.3322/caac.21820 38230766

[2] Sanderson K (2023) Huge leap in breast cancer survival rate. Nature. 10.1038/d41586-023-02075-w 37353635 10.1038/d41586-023-02075-w

[3] Wickerham DL et al (2008) The half century of clinical trials of the national surgical adjuvant breast and bowel project. Semin Oncol 35(5):522–529 18929150 10.1053/j.seminoncol.2008.07.005 PMC2583142

[4] Harbeck N et al (2019) Breast cancer. Nat Rev Dis Primers 5(1):66 31548545 10.1038/s41572-019-0111-2

[5] Fisher B et al (2002) Twenty-year follow-up of a randomized trial comparing total mastectomy, lumpectomy, and lumpectomy plus irradiation for the treatment of invasive breast cancer. N Engl J Med 347(16):1233–1241 12393820 10.1056/NEJMoa022152

[6] Clarke M et al (2005) Effects of radiotherapy and of differences in the extent of surgery for early breast cancer on local recurrence and 15-year survival: an overview of the randomised trials. Lancet 366(9503):2087–2106 16360786 10.1016/S0140-6736(05)67887-7

[7] McGale P et al (2014) Effect of radiotherapy after mastectomy and axillary surgery on 10-year recurrence and 20-year breast cancer mortality: meta-analysis of individual patient data for 8135 women in 22 randomised trials. Lancet 383(9935):2127–2135 24656685 10.1016/S0140-6736(14)60488-8 PMC5015598

[8] Cardoso F et al (2017) European breast cancer conference manifesto on breast centres/units. Eur J Cancer 72:244–250 28064097 10.1016/j.ejca.2016.10.023

[9] Pasquier D et al (2023) Designing clinical trials based on modern imaging and metastasis-directed treatments in patients with oligometastatic breast cancer: a consensus recommendation from the EORTC imaging and breast cancer groups. Lancet Oncol 24(8):e331–e343 37541279 10.1016/S1470-2045(23)00286-3

[10] Goldberg M et al (2023) A meta-analysis of trials of partial breast irradiation. Int J Radiat Oncol Biol Phys 115(1):60–72 36155214 10.1016/j.ijrobp.2022.09.062

[11] Strnad V et al (2023) Accelerated partial breast irradiation using sole interstitial multicatheter brachytherapy compared with whole-breast irradiation with boost for early breast cancer: 10-year results of a GEC-ESTRO randomised, phase 3, non-inferiority trial. Lancet Oncol 24(3):262–272 36738756 10.1016/S1470-2045(23)00018-9

[12] Shaitelman SF et al (2024) Partial breast irradiation for patients with early-stage invasive breast cancer or ductal carcinoma in situ: an ASTRO clinical practice guideline. Pract Radiat Oncol 14(2):112–132 37977261 10.1016/j.prro.2023.11.001

[13] Shickh S et al (2023) Shared decision making in the care of patients with cancer. Am Soc Clin Oncol Educ Book 43:e389516 37339391 10.1200/EDBK_389516

[14] https://www.nccn.org/login?ReturnURL=https://www.nccn.org/professionals/physician_gls/pdf/breast.pdf

[15] Curigliano G et al (2023) Understanding breast cancer complexity to improve patient outcomes: the St Gallen international consensus conference for the primary therapy of individuals with early breast cancer 2023. Ann Oncol 34(11):970–986 37683978 10.1016/j.annonc.2023.08.017

[16] Loibl S et al (2024) Early breast cancer: ESMO clinical practice guideline for diagnosis, treatment and follow-up. Ann Oncol 35(2):159–182 38101773 10.1016/j.annonc.2023.11.016

[17] Brackstone M et al (2021) Management of the axilla in early-stage breast cancer: Ontario health (cancer care Ontario) and ASCO guideline. J Clin Oncol 39(27):3056–3082 34279999 10.1200/JCO.21.00934

[18] Morrow M et al (2016) Society of surgical oncology-American society for radiation oncology-American society of clinical oncology consensus guideline on margins for breast-conserving surgery with whole-breast irradiation in ductal carcinoma in situ. Pract Radiat Oncol 6(5):287–295 27538810 10.1016/j.prro.2016.06.011 PMC5070537

[19] Recht A et al (2016) Postmastectomy radiotherapy: an American society of clinical oncology, American society for radiation oncology, and society of surgical oncology focused guideline update. J Clin Oncol 34(36):4431–4442 27646947 10.1200/JCO.2016.69.1188

[20] Fastner G et al (2020) ESTRO IORT task force/ACROP recommendations for intraoperative radiation therapy with electrons (IOERT) in breast cancer. Radiother Oncol 149:150–157 32413529 10.1016/j.radonc.2020.04.059

[21] Strnad V et al (2018) ESTRO-ACROP guideline: interstitial multi-catheter breast brachytherapy as accelerated partial breast irradiation alone or as boost—GEC-ESTRO breast cancer working group practical recommendations. Radiother Oncol 128(3):411–420 29691075 10.1016/j.radonc.2018.04.009

[22] Meattini I et al (2022) European society for radiotherapy and oncology advisory committee in radiation oncology practice consensus recommendations on patient selection and dose and fractionation for external beam radiotherapy in early breast cancer. Lancet Oncol 23(1):e21–e31 34973228 10.1016/S1470-2045(21)00539-8

[23] Meattini I et al (2024) International multidisciplinary consensus on the integration of radiotherapy with new systemic treatments for breast cancer: European society for radiotherapy and oncology (ESTRO)-endorsed recommendations. Lancet Oncol 25(2):e73–e83 38301705 10.1016/S1470-2045(23)00534-X

[24] EBCTCG (Early Breast Cancer Trialists’ Collaborative Group) (2023) Radiotherapy to regional nodes in early breast cancer: an individual patient data meta-analysis of 14 324 women in 16 trials. Lancet 402(10416):1991–2003 37931633 10.1016/S0140-6736(23)01082-6

[25] Kaidar-Person O et al (2023) The Lucerne toolbox 2 to optimise axillary management for early breast cancer: a multidisciplinary expert consensus. eClinicalMedicine. 10.1016/j.eclinm.2023.102085 37528842 10.1016/j.eclinm.2023.102085 PMC10388578

[26] Giuliano AE et al (2017) Effect of axillary dissection vs no axillary dissection on 10-year overall survival among women with invasive breast cancer and sentinel node metastasis: the ACOSOG Z0011 (alliance) randomized clinical trial. JAMA 318(10):918–926 28898379 10.1001/jama.2017.11470 PMC5672806

[27] Donker M et al (2014) Radiotherapy or surgery of the axilla after a positive sentinel node in breast cancer (EORTC 10981-22023 AMAROS): a randomised, multicentre, open-label, phase 3 non-inferiority trial. Lancet Oncol 15(12):1303–1310 25439688 10.1016/S1470-2045(14)70460-7 PMC4291166

[28] Whelan TJ et al (2015) Regional nodal irradiation in early-stage breast cancer. N Engl J Med 373(4):307–316 26200977 10.1056/NEJMoa1415340 PMC4556358

[29] Poortmans PM et al (2015) Internal mammary and medial supraclavicular irradiation in breast cancer. N Engl J Med 373(4):317–327 26200978 10.1056/NEJMoa1415369

[30] Poortmans P (2014) Postmastectomy radiation in breast cancer with one to three involved lymph nodes: ending the debate. Lancet 383(9935):2104–2106 24656686 10.1016/S0140-6736(14)60192-6

[31] de Wild SR et al (2022) De-escalation of radiotherapy after primary chemotherapy in cT1-2N1 breast cancer (RAPCHEM; BOOG 2010-03): 5‑year follow-up results of a Dutch, prospective, registry study. Lancet Oncol 23(9):1201–1210 35952707 10.1016/S1470-2045(22)00482-X

[32] Mamounas E, Bandos H, White J et al (2023) Loco-regional irradiation in patients with biopsy-proven axillary node involvement at presentation who become pathologically node-negative after neoadjuvant chemotherapy: primary outcomes of NRG oncology/NSABP B‑51/RTOG 1304. 2023 San Antonio Breast Cancer Symposium. (Abstract GS02-07)

[33] Gentilini OD et al (2023) Sentinel lymph node biopsy vs no axillary surgery in patients with small breast cancer and negative results on ultrasonography of axillary lymph nodes: the SOUND randomized clinical trial. JAMA Oncol 9(11):1557–1564 37733364 10.1001/jamaoncol.2023.3759 PMC10514873

[34] https://ascopubs.org/jco/special-series/2020/local-regional-management-breast-cancer

[35] Chua BH et al (2022) Radiation doses and fractionation schedules in non-low-risk ductal carcinoma in situ in the breast (BIG 3‑07/TROG 07.01): a randomised, factorial, multicentre, open-label, phase 3 study. Lancet 400(10350):431–440 35934006 10.1016/S0140-6736(22)01246-6

[36] Vicini FA et al (2022) NRG RTOG 1005: a phase III trial of hypo fractionated whole breast irradiation with concurrent boost vs. conventional whole breast irradiation plus sequential boost following lumpectomy for high risk early-stage breast cancer. Int J Radiat Oncol Biol Phys 114(3):S1

[37] Meattini I et al (2020) Accelerated partial-breast irradiation compared with whole-breast irradiation for early breast cancer: long-term results of the randomized phase III APBI-IMRT-florence trial. J Clin Oncol 38(35):4175–4183 32840419 10.1200/JCO.20.00650

[38] Brunt AM et al (2020) Ten-year results of FAST: a randomized controlled trial of 5‑fraction whole-breast radiotherapy for early breast cancer. J Clin Oncol 38(28):3261–3272 32663119 10.1200/JCO.19.02750 PMC7526720

[39] Murray Brunt A et al (2020) Hypofractionated breast radiotherapy for 1 week versus 3 weeks (FAST-Forward): 5‑year efficacy and late normal tissue effects results from a multicentre, non-inferiority, randomised, phase 3 trial. Lancet 395(10237):1613–1626 32580883 10.1016/S0140-6736(20)30932-6 PMC7262592

[40] Aznar MC et al (2023) ESTRO-ACROP guideline: recommendations on implementation of breath-hold techniques in radiotherapy. Radiother Oncol 185:109734 37301263 10.1016/j.radonc.2023.109734

[41] Kron T et al (2022) TROG 14.04: multicentre study of feasibility and impact on anxiety of DIBH in breast cancer patients. Clin Oncol 34(9):e410–e419 10.1016/j.clon.2022.05.020 35717318

[42] Vesprini D et al (2022) Effect of supine vs prone breast radiotherapy on acute toxic effects of the skin among women with large breast size: a randomized clinical trial. JAMA Oncol 8(7):994–1000 35616948 10.1001/jamaoncol.2022.1479 PMC9136674

[43] Kaidar-Person O et al (2021) A Delphi study and international consensus recommendations: the use of bolus in the setting of postmastectomy radiation therapy for early breast cancer. Radiother Oncol 164:115–121 34563607 10.1016/j.radonc.2021.09.012

[44] Offersen BV et al (2015) ESTRO consensus guideline on target volume delineation for elective radiation therapy of early stage breast cancer. Radiother Oncol 114(1):3–10 25630428 10.1016/j.radonc.2014.11.030

[45] Jing H et al (2023) Individualized clinical target volume for irradiation of the supraclavicular region in breast cancer based on mapping of the involved ipsilateral supraclavicular lymph nodes. Int J Radiat Oncol Biol Phys 115(4):922–932 36368434 10.1016/j.ijrobp.2022.10.030

[46] https://www.rtog.org/LinkClick.aspx?fileticket=eVB451KQ83M%3d&tabid=429

[47] Kaidar-Person O et al (2019) ESTRO ACROP consensus guideline for target volume delineation in the setting of postmastectomy radiation therapy after implant-based immediate reconstruction for early stage breast cancer. Radiother Oncol 137:159–166 31108277 10.1016/j.radonc.2019.04.010

[48] Owen JR et al (2006) Effect of radiotherapy fraction size on tumour control in patients with early-stage breast cancer after local tumour excision: long-term results of a randomised trial. Lancet Oncol 7(6):467–471 16750496 10.1016/S1470-2045(06)70699-4

[49] Bentzen SM et al (2008) The UK standardisation of breast radiotherapy (START) trial A of radiotherapy hypofractionation for treatment of early breast cancer: a randomised trial. Lancet Oncol 9(4):331–341 18356109 10.1016/S1470-2045(08)70077-9 PMC2323709

[50] Bentzen SM et al (2008) The UK standardisation of breast radiotherapy (START) trial B of radiotherapy hypofractionation for treatment of early breast cancer: a randomised trial. Lancet 371(9618):1098–1107 18355913 10.1016/S0140-6736(08)60348-7 PMC2277488

[51] Whelan TJ et al (2010) Long-term results of hypofractionated radiation therapy for breast cancer. N Engl J Med 362(6):513–520 20147717 10.1056/NEJMoa0906260

[52] Coles CE et al (2017) Partial-breast radiotherapy after breast conservation surgery for patients with early breast cancer (UK IMPORT LOW trial): 5‑year results from a multicentre, randomised, controlled, phase 3, non-inferiority trial. Lancet 390(10099):1048–1060 28779963 10.1016/S0140-6736(17)31145-5 PMC5594247

[53] Wang SL et al (2019) Hypofractionated versus conventional fractionated postmastectomy radiotherapy for patients with high-risk breast cancer: a randomised, non-inferiority, open-label, phase 3 trial. Lancet Oncol 20(3):352–360 30711522 10.1016/S1470-2045(18)30813-1

[54] Wang S‑L et al Hypofractionated versus conventional fractionated radiotherapy after breast-conserving surgery in the modern treatment era: a multicenter, randomized controlled trial from China. J Clin Oncol. 10.1200/jco.20.01024 10.1200/JCO.20.01024 32780661

[55] Offersen BV et al Hypofractionated versus standard fractionated radiotherapy in patients with early breast cancer or ductal carcinoma in situ in a randomized phase III trial: the DBCG HYPO trial. J Clin Oncol. 10.1200/jco.20.01363 10.1200/JCO.20.01363 32910709

[56] Murray Brunt A et al (2020) Hypofractionated breast radiotherapy for 1 week versus 3 weeks (FAST-Forward): 5‑year efficacy and late normal tissue effects results from a multicentre, non-inferiority, randomised, phase 3 trial. Lancet 395(10237):1613–1626 32580883 10.1016/S0140-6736(20)30932-6 PMC7262592

[57] Coles CE et al (2023) Dose-escalated simultaneous integrated boost radiotherapy in early breast cancer (IMPORT HIGH): a multicentre, phase 3, non-inferiority, open-label, randomised controlled trial. Lancet 401(10394):2124–2137 37302395 10.1016/S0140-6736(23)00619-0

[58] Kunkler IH et al (2015) Breast-conserving surgery with or without irradiation in women aged 65 years or older with early breast cancer (PRIME II): a randomised controlled trial. Lancet Oncol 16(3):266–273 25637340 10.1016/S1470-2045(14)71221-5

[59] Whelan TJ et al (2023) Omitting radiotherapy after breast-conserving surgery in luminal A breast cancer. N Engl J Med 389(7):612–619 37585627 10.1056/NEJMoa2302344

[60] Jagsi R et al (2024) Omission of radiotherapy after breast-conserving surgery for women with breast cancer with low clinical and genomic risk: 5‑year outcomes of IDEA. J Clin Oncol 42(4):390–398 38060195 10.1200/JCO.23.02270 PMC11846025

[61] Ho AY, Bellon JR (2023) Overcoming resistance—omission of radiotherapy for low-risk breast cancer. N Engl J Med 388(7):652–653 36791166 10.1056/NEJMe2216133

[62] Recht A (2023) Omitting radiotherapy in luminal A breast cancer. N Engl J Med 389(18):1727 37913518 10.1056/NEJMc2310656

[63] Ward MC et al (2019) Radiation therapy without hormone therapy for women age 70 or above with low-risk early breast cancer: a microsimulation. Int J Radiat Oncol Biol Phys 105(2):296–306 31212043 10.1016/j.ijrobp.2019.06.014

[64] https://clinicaltrials.gov/study/NCT04134598?cond=breast&term=europa&rank=1

[65] Purswani JM et al (2021) Breast conservation in women with autoimmune disease: the role of active autoimmune disease and hypofractionation on acute and late toxicity in a case-controlled series. Int J Radiat Oncol Biol Phys 110(3):783–791 33545303 10.1016/j.ijrobp.2021.01.047

[66] Purswani JM et al (2022) Toxicity and cosmetic outcome of breast irradiation in women with breast cancer and autoimmune connective tissue disease: the role of fraction and field size. Pract Radiat Oncol 12(2):e90–e100 34774868 10.1016/j.prro.2021.10.008

[67] Thariat J et al (2021) Avoidance or adaptation of radiotherapy in patients with cancer with Li-Fraumeni and heritable TP53-related cancer syndromes. Lancet Oncol 22(12):e562–e574 34856153 10.1016/S1470-2045(21)00425-3

[68] Turnbull C, Mirugaesu N, Eeles R (2006) Radiotherapy and genetic predisposition to breast cancer. Clin Oncol 18(3):257–267 10.1016/j.clon.2005.11.013 16605057

[69] Chapman BV et al (2022) Breast radiation therapy-related treatment outcomes in patients with or without germline mutations on multigene panel testing. Int J Radiat Oncol Biol Phys 112(2):437–444 34582940 10.1016/j.ijrobp.2021.09.026 PMC8748284

[70] Kirova YM et al (2005) Risk of breast cancer recurrence and contralateral breast cancer in relation to BRCA 1 and BRCA 2 mutation status following breast-conserving surgery and radiotherapy. Eur J Cancer 41(15):2304–2311 16140006 10.1016/j.ejca.2005.02.037

[71] Pierce L (2002) Radiotherapy for breast cancer in BRCA 1/BRCA 2 carriers: clinical issues and management dilemmas. Semin Radiat Oncol 12(4):352–361 12382193 10.1053/srao.2002.35254

[72] Kirova YM et al (2010) Is the breast-conserving treatment with radiotherapy appropriate in BRCA 1/2 mutation carriers? Long-term results and review of the literature. Breast Cancer Res Treat 120(1):119–126 20033769 10.1007/s10549-009-0685-6

[73] Meattini I et al (2022) Integrating radiation therapy with targeted treatments for breast cancer: from bench to bedside. Cancer Treat Rev 108:102417 35623219 10.1016/j.ctrv.2022.102417

[74] Halyard MY et al (2009) Radiotherapy and adjuvant trastuzumab in operable breast cancer: tolerability and adverse event data from the NCCTG phase III trial N9831. J Clin Oncol 27(16):2638–2644 19349549 10.1200/JCO.2008.17.9549 PMC2690390

[75] Debbi K et al (2023) Interaction between radiation therapy and targeted therapies in HER2-positive breast cancer: literature review, levels of evidence for safety and recommendations for optimal treatment sequence. Cancers. 10.3390/cancers15082278 37190205 10.3390/cancers15082278 PMC10137001

[76] Salvestrini V et al (2023) Safety profile of trastuzumab-emtansine (T-DM1) with concurrent radiation therapy: a systematic review and meta-analysis. Radiother Oncol 186:109805 37437610 10.1016/j.radonc.2023.109805

[77] Verma S et al (2024) Immunotherapy and radiation therapy sequencing in breast cancer: a systematic review. Int J Radiat Oncol Biol Phys 118(5):1422–1434 38195030 10.1016/j.ijrobp.2024.01.001

[78] Visani L et al (2022) Safety of CDK4/6 inhibitors and concomitant radiation therapy in patients affected by metastatic breast cancer. Radiother Oncol 177:40–45 36349599 10.1016/j.radonc.2022.10.023

[79] Loap P et al (2022) Concurrent olaparib and radiotherapy in patients with triple-negative breast cancer: the phase 1 olaparib and radiation therapy for triple-negative breast cancer trial. JAMA Oncol 8(12):1802–1808 36301572 10.1001/jamaoncol.2022.5074 PMC9614672

[80] Tutt ANJ et al (2021) Adjuvant olaparib for patients with 〈i〉BRCA 1〈/i〉- or 〈i〉BRCA 2〈/i〉-mutated breast cancer. N Engl J Med 384(25):2394–2405 34081848 10.1056/NEJMoa2105215 PMC9126186

[81] Woodward WA et al (2017) A phase 2 study of capecitabine and concomitant radiation in women with advanced breast cancer. Int J Radiat Oncol Biol Phys 99(4):777–783 28843370 10.1016/j.ijrobp.2017.04.030 PMC6072264

[82] Masuda N et al (2017) Adjuvant capecitabine for breast cancer after preoperative chemotherapy. N Engl J Med 376(22):2147–2159 28564564 10.1056/NEJMoa1612645

[83] Kroeze SGC et al (2023) Metastases-directed stereotactic body radiotherapy in combination with targeted therapy or immunotherapy: systematic review and consensus recommendations by the EORTC-ESTRO OligoCare consortium. Lancet Oncol 24(3):e121–e132 36858728 10.1016/S1470-2045(22)00752-5

[84] Abeloos CH et al (2023) Different re-irradiation techniques after breast-conserving surgery for recurrent or new primary breast cancer. Curr Oncol 30(1):1151–1163 36661737 10.3390/curroncol30010088 PMC9857440

[85] Fattahi S et al (2021) Reirradiation for locoregional recurrent breast cancer. Adv Radiat Oncol. 10.1016/j.adro.2020.100640 33506143 10.1016/j.adro.2020.100640 PMC7814100

[86] Hannoun-Levi JM et al (2021) Salvage mastectomy versus second conservative treatment for second Ipsilateral breast tumor event: a propensity score-matched cohort analysis of the GEC-ESTRO breast cancer working group database. Int J Radiat Oncol Biol Phys 110(2):452–461 33383125 10.1016/j.ijrobp.2020.12.029

[87] Arthur DW et al (2020) Effectiveness of breast-conserving surgery and 3‑dimensional conformal partial breast reirradiation for recurrence of breast cancer in the Ipsilateral breast: the NRG oncology/RTOG 1014 phase 2 clinical trial. JAMA Oncol 6(1):75–82 31750868 10.1001/jamaoncol.2019.4320 PMC6902101

[88] Chen I et al (2021) Salvage of locally recurrent breast cancer with repeat breast conservation using 45 Gy hyperfractionated partial breast re-irradiation. Breast Cancer Res Treat 188(2):409–414 33770311 10.1007/s10549-021-06206-7

[89] Hannoun-Levi JM et al (2013) Accelerated partial breast irradiation with interstitial brachytherapy as second conservative treatment for ipsilateral breast tumour recurrence: multicentric study of the GEC-ESTRO breast cancer working group. Radiother Oncol 108(2):226–231 23647758 10.1016/j.radonc.2013.03.026

[90] Montagne L, Hannoun A, Hannoun-Levi J‑M (2020) Second conservative treatment for second ipsilateral breast tumor event: a systematic review of the different re-irradiation techniques. Breast 49:274–280 31945697 10.1016/j.breast.2020.01.003 PMC7375668

[91] Kaidar-Person O, Oldenborg S, Poortmans P (2018) Re-irradiation and hyperthermia in breast cancer. Clin Oncol 30(2):73–84 10.1016/j.clon.2017.11.004 29224899

[92] Notter M et al (2020) Combined wIRA-hyperthermia and hypofractionated re-irradiation in the treatment of locally recurrent breast cancer: evaluation of therapeutic outcome based on a novel size classification. Cancers. 10.3390/cancers12030606 32155740 10.3390/cancers12030606 PMC7139693

[93] Andratschke N et al (2022) European society for radiotherapy and oncology and European organisation for research and treatment of cancer consensus on re-irradiation: definition, reporting, and clinical decision making. Lancet Oncol 23(10):e469–e478 36174633 10.1016/S1470-2045(22)00447-8

[94] Andersen BL et al (2023) Management of anxiety and depression in adult survivors of cancer: ASCO guideline update. J Clin Oncol 41(18):3426–3453 37075262 10.1200/JCO.23.00293

[95] Carlson LE et al (2023) Integrative oncology care of symptoms of anxiety and depression in adults with cancer: society for integrative oncology—ASCO guideline. J Clin Oncol 41(28):4562–4591 37582238 10.1200/JCO.23.00857

[96] Bower JE et al (2014) Screening, assessment, and management of fatigue in adult survivors of cancer: an American society of clinical oncology clinical practice guideline adaptation. J Clin Oncol 32(17):1840–1850 24733803 10.1200/JCO.2013.53.4495 PMC4039870

[97] Naoum GE, Taghian AG (2023) Endocrine Treatment for 5 Years or Radiation for 5 Days for Patients With Early Breast Cancer Older Than 65 Years: Can We Do It Right? J Clin Oncol 41(13):2331–2336. 10.1200/JCO.22.02171 36538740 10.1200/JCO.22.02171

